# Correlation between Ultrasound Peak Systolic Velocity and Angiography for Grading Internal Carotid Artery Stenosis

**DOI:** 10.3390/jcm13020517

**Published:** 2024-01-17

**Authors:** Dan-Alexandru Tătaru, Maria Olinic, Călin Homorodean, Mihai-Claudiu Ober, Mihail Spînu, Florin-Leontin Lazăr, Laurențiu Onea, Dan-Mircea Olinic

**Affiliations:** 1 Medical Clinic No. 1, University of Medicine and Pharmacy “Iuliu Hatieganu”, 400006 Cluj-Napoca, Romania; tataru.cardio@gmail.com (D.-A.T.); chomorodean@yahoo.com (C.H.); spinu_mihai@yahoo.com (M.S.); lazar.leontin@yahoo.com (F.-L.L.); onea.lau91@yahoo.com (L.O.); danolinic@gmail.com (D.-M.O.); 2 Interventional Cardiology Department, Cluj County Emergency Hospital, 400006 Cluj-Napoca, Romania; mihai_ober@yahoo.com

**Keywords:** carotid artery disease, peak systolic velocity, digital subtraction angiography

## Abstract

(1) Background: The success of carotid revascularization depends on the accurate grading of carotid stenoses. Therefore, it is important for every vascular center to establish its protocols for the same. In this study, we aimed to determine the peak systolic velocity (PSV) thresholds that can predict moderate and severe internal carotid artery (ICA) stenoses. (2) Methods: To achieve this, we enrolled patients who underwent both duplex ultrasound (DUS) and invasive carotid artery digital subtraction angiography (DSA). The degree of ICA stenosis was assessed using the North American Symptomatic Carotid Endarterectomy Trial (NASCET) and the European Carotid Surgery Trial (ECST) protocols. The PSV thresholds were determined using receiver operating characteristic (ROC) curves. (3) Results: Our study included 47 stenoses, and we found that the PSV cut-off for predicting ≥70% NASCET ICA stenoses was 200 cm/s (sensitivity 90.32%, specificity 93.75%). However, PSV did not correlate significantly with ≥50% NASCET ICA stenoses. On the other hand, the optimal PSV threshold for predicting ≥80% ECST ICA stenoses was 180 cm/s (sensitivity 100%, specificity 81.82%). (4) Conclusions: Based on our findings, we concluded that PSV is a good and simple marker for the identification of severe stenoses. We found that PSV values correlate significantly with severe NASCET and ECST stenoses, with 200 cm/s and 180 cm/s PSV thresholds. However, PSV was not reliable with moderate NASCET stenoses. In such cases, complementary imaging should be used.

## 1. Introduction

Stroke is the second leading cause of disability and death worldwide, with low- and middle-income countries bearing the highest burden of the disease [1]. Ischemic strokes account for 87% of all strokes. In a European population of 715 million, about 1.4 million strokes occur each year [2]. Carotid artery disease, along with cardiac embolism, is one of the most common causes of cerebrovascular accidents [3,4]. Atherosclerosis generally affects the carotid bifurcation or the proximal segment of the internal carotid artery (ICA). Prompt evaluation of the carotid stenosis is crucial to determine whether the patient can benefit from revascularization [5].

Several randomized clinical trials have shown the efficacy of carotid endarterectomy (CEA) in treating selected ICA stenoses [6,7,8,9]. A large meta-analysis comparing the two revascularization techniques has also suggested that carotid artery stenting (CAS) can be beneficial for patients at high surgical risk [10]. Duplex ultrasound (DUS) is the preferred method for screening and diagnosing carotid artery disease. It is a non-ionizing, widely available, and repeatable method that provides valuable information on vessel anatomy. However, it is operator-dependent and has considerable variability in the equipment and the criteria used for stenosis assessment. The accuracy of DUS may be limited by technical errors, additional stenotic lesions, inability to distinguish pseudo-occlusion, underestimation of highly calcified plaques, and several other issues [5].

Measuring peak systolic velocity (PSV) is a crucial part of the DUS examination and is also straightforward to perform [11]. PSV increases as the artery narrows, except for pre-occlusive lesions, where PSV tends to decrease [12]. While other factors such as the ICA-to-common carotid artery (CCA) PSV ratio, end-diastolic velocity (EDV), and caliper measurements provide additional useful information for determining significant plaques [12,13], PSV remains the most effective ultrasound parameter for detecting carotid stenosis [14]. Additionally, PSV is easy to obtain and has good intra- and inter-observer reproducibility [15]. It is now widely accepted that individual laboratories should validate the velocity criteria instead of adopting reported criteria from other laboratories [5] or current guidelines [3,12,16]. Rigid PSV cut-offs applied in one major clinical trial had moderate sensitivity and specificity (65 to 71%) for grading carotid stenoses [6]. Therefore, each vascular center must assess the accuracy of the DUS examination compared to the ‘gold standard’ so that the maximum benefit of carotid revascularization can be achieved. Despite the advances in non-invasive imaging techniques, digital subtraction angiography (DSA) is still the ‘gold standard’ for the diagnosis of carotid artery disease. However, it is an invasive procedure that could cause peri-operative complications. Moreover, it yields high X-ray exposure for both patients and personnel [17].

This study aimed to correlate the PSV values found ‘in-house’ with the degree of carotid stenoses described by DSA.

## 2. Materials and Methods

Between August 2018 and December 2021, our invasive center received patient referrals from neurologists, cardiologists, and vascular surgeons. This study focused on carotid artery stenosis, which refers to a narrowing of the extra-cranial part of the ICA. The first procedure performed on each patient in the study was a DUS examination with PSV measurement. Both carotid arteries were examined by two clinicians with over 10 years of experience in vascular ultrasound. DUS was performed with a General Electric Vivid Q ultrasound machine (GE HealthCare, Chicago, IL, USA). A linear probe with an emitting frequency range of 4 to 7 MHz was used (GE HealthCare). The carotid plaque was first identified on the B-mode scan, and the transducer was then placed longitudinally, parallel to the carotid artery, on the neck. The angle-corrected velocity measurement was performed to obtain the stenotic jet velocity spectrum at the origin of the ICA. Patients with complex stenoses involving the common carotid artery or occluded vessels were excluded.

**Figure 1:**
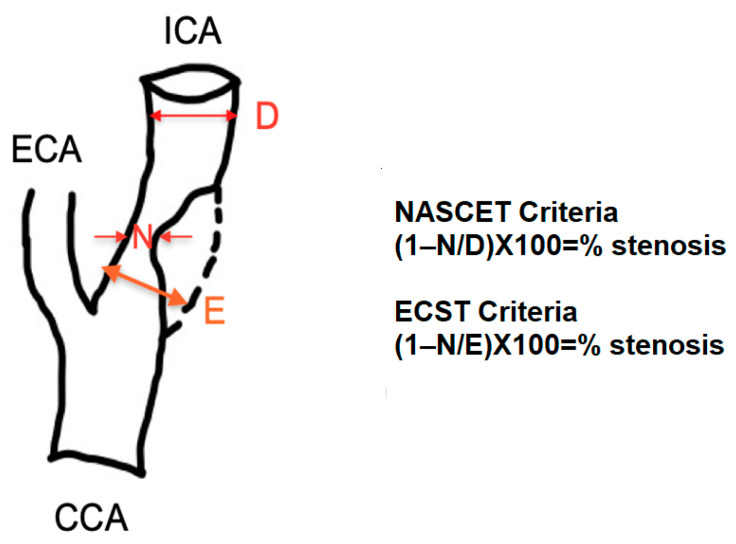
ICA stenosis calculation after NASCET [6] and ECST [7] methods. Abbreviations: NASCET, North American Symptomatic Carotid Endarterectomy Trial; ECST, European Carotid Surgery Trial; CCA, common carotid artery; ECA, external carotid artery; ICA, internal carotid artery; N, minimum lumen diameter; D, vertical segment diameter; E, estimated bulb diameter.

Patients with a PSV value above 125 cm/s in the proximal ICA and with visible plaque were selected for invasive DSA. The 125 cm/s PSV threshold was chosen based on current guidelines for selecting ICA stenoses >50%. All patients referred to undertake invasive DSA were candidates for ICA revascularization, such as recent ischemic stroke or transient ischemic attack, heart surgery referral, or high-risk patients for future ischemic events. Invasive DSA was performed in the same hospitalization, after DUS screening. DSA images were obtained with a Siemens Artis Zee floor-standing angiograph equipped with a digital subtraction feature (Siemens AG, Berlin, Germany). Selective bilateral common carotid injections were performed in the anteroposterior and lateral projections. The measurements were made by two experienced invasive cardiologists and expressed as a percentage (%) of the diameter reduction of the vessel. Lesions were noted with an increment of 10%. The percentage of the tightest stenosis was noted for statistical analysis. Two protocols were used for angiographic stenosis quantification, namely the North American Symptomatic Carotid Endarterectomy Trial (NASCET) and the European Carotid Surgery Trial (ECST) criteria. The NASCET protocol considers the baseline diameter of the first segment of the far vertical ICA with parallel walls, beyond the post-stenotic dilatation. NASCET ICA stenoses were classified as ≥50%, ≥60%, and ≥70%. Near-occluded vessels, based on the angiographic collapse of the vertical segment of the ICA, were excluded. The ECST protocol estimates the real diameter of the ICA bulb and calculates the stenosis percentage accordingly. ECST stenoses were classified as severe if ≥80%. The protocols are depicted in Figure 1. DUS and DSA results were acquired independently.

All patients provided informed consent upon admission and before the invasive DSA procedure, granting permission for publication. This study was approved by the hospital’s ethical committee and conducted in accordance with the Declaration of Helsinki. Statistical analysis was performed using MedCalc software (version 19.2.6) from MedCalc Software Ltd., Ostend, Belgium. Receiver operating characteristic (ROC) curves and the Youden index were applied for analysis. ROC analysis was used to select optimal models and discard suboptimal ones, independently. The degree or measure of separability was represented by the area under the ROC curve (AUC). AUC values of 0.9–1 were considered excellent, 0.8–0.9 good, 0.7–0.8 fair, 0.6–0.7 poor, and 0.5–0.6 failed [18]. The Youden index was used to capture the performance of a diagnostic test and was often used in conjunction with ROC analysis. An acceptable Youden index cut-off point was 0.5, with any value below indicating an overall lack of the diagnostic test to detect either disease or health [19,20]. A *p*-value less than 0.05 was considered significant.

## 3. Results

This study analyzed 35 patients who underwent DUS and DSA. The patients had a mean age of 65.8 ± 14.2 years, with 77% males and 23% females. The mean interval between DSA and DUS was 2 ± 1 days. The study included 47 carotid lesions, out of which 28 were symptomatic (59.5%). The mean stenosis was 68.92 ± 16.18% (median, 70%; range, 60%) with the NASCET method and 84.80 ± 8.56% (median, 85%; range, 32%) with the ECST method. The mean PSV was 263 ± 93 cm/s (median, 250 cm/s; range, 400 cm/s).

### 3.1. NASCET Criteria

**Figure 2:**
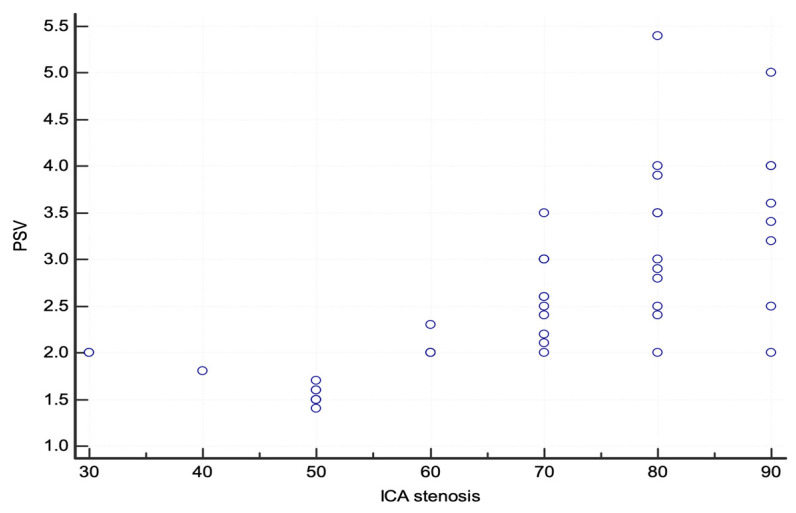
The scatter plot of PSV and the degree of internal carotid artery stenosis calculated using the NASCET method on invasive angiography. Abbreviations: NASCET, North American Symptomatic Carotid Endarterectomy Trial [6]; PSV, peak systolic velocity.

According to the NASCET criteria, three lesions were <50%, seven lesions were 50–59%, five lesions were 60–69%, and the remaining thirty lesions were ≥70%. The scatter plot of PSV and the degree of ICA stenosis is shown in Figure 2.

**Figure 3:**
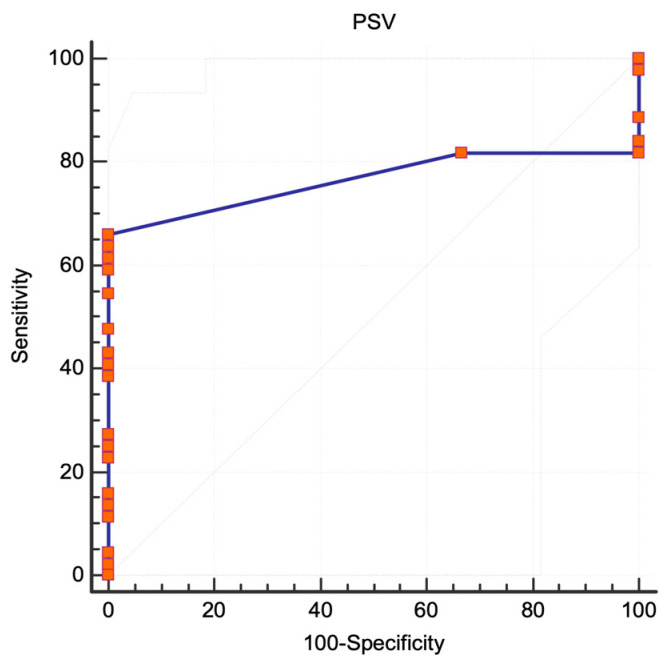
NASCET criteria, ROC analysis. The area under the curve for stenoses ≥50%. Abbreviations: NASCET, North American Symptomatic Carotid Endarterectomy Trial [6]; ROC, receiver operating characteristic; PSV, peak systolic velocity.

For ICA stenoses ≥50%, PSV was not an accurate selection method, with a fair selection expressing an AUC of 0.765 (95% CI 0.619 to 0.876, standard error 0.063, *p* = 0.0001) and a modest power of the diagnostic test expressed by the Youden index of 0.65, as depicted in Figure 3.

**Figure 4:**
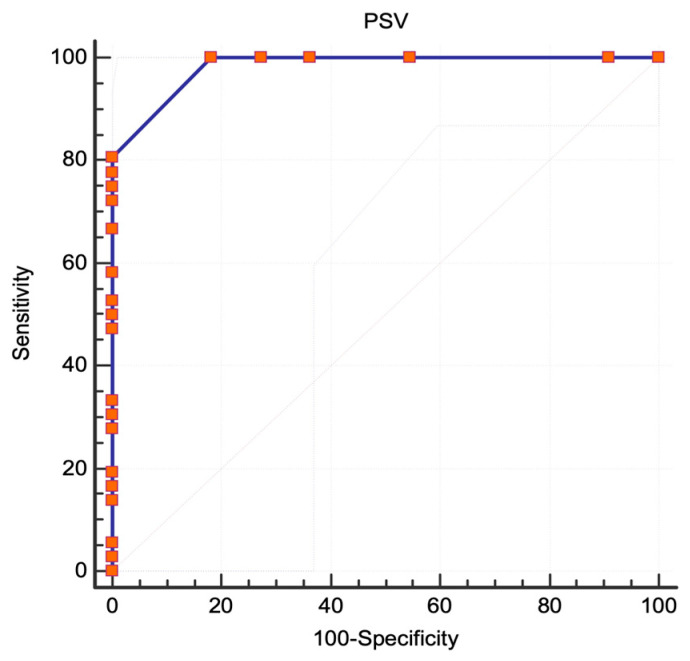
NASCET criteria, ROC analysis. The area under the curve for stenoses ≥60%; Abbreviations: NASCET, North American Symptomatic Carotid Endarterectomy Trial [6]; ROC, receiver operating characteristic; PSV, peak systolic velocity.

However, for ICA stenoses ≥60%, PSV was a good criterion for selection, with an excellent correlation expressed by an AUC of 0.982 (95% CI 0.893 to 1, standard error 0.0133, *p* < 0.0001) (Figure 4). The best-associated criterion was a PSV threshold of 180 cm/s, with a sensitivity of 100% (95% CI 90.3–100) and a specificity of 81.82% (95% CI 48.2–97.7), and the Youden index of 0.81 suggested a good performance of the test. The sensitivity and specificity of various PSV thresholds at intervals of 10 cm/s are presented in Table 1.

**Figure 5:**
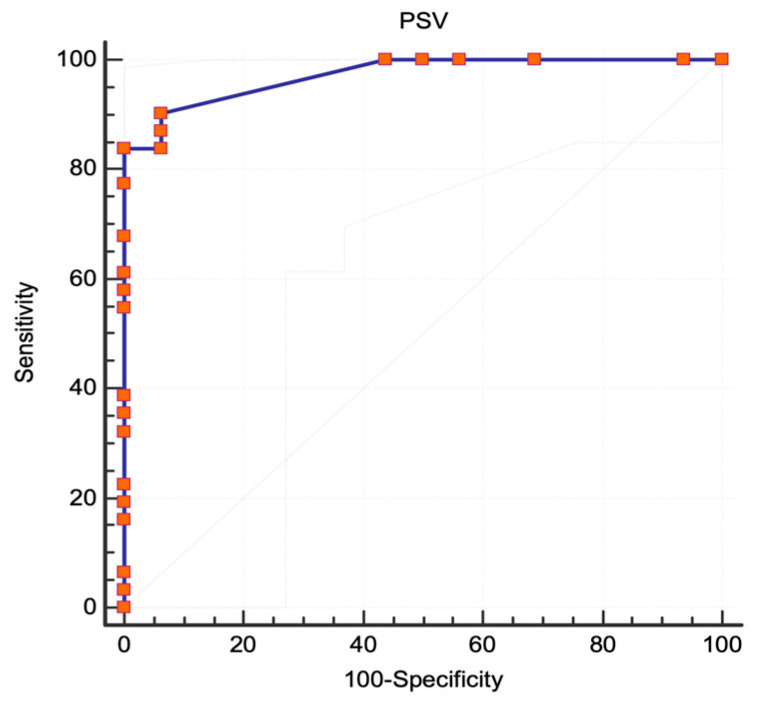
NASCET criteria, ROC analysis. The area under the curve for stenoses ≥70%; Abbreviations: NASCET, North American Symptomatic Carotid Endarterectomy Trial [6]; ROC, receiver operating characteristic; PSV, peak systolic velocity.

For severe lesions (≥70%) of the ICA, DUS was highly accurate, with an AUC of 0.972 (95% CI 0.876 to 0.998, *p* < 0.0001) (Figure 5). The best-associated criterion was a PSV threshold of 200 cm/s, with a sensitivity of 90.32% (95% CI 74.2–98.0) and a specificity of 93.75% (95% CI 69.8–99.8). The 230 cm/s PSV cut-off had a sensitivity of 83.87% (95% CI 66.3–94.5) and a specificity of 100% (95% CI 79.4–100). The sensitivity and specificity of various PSV thresholds at intervals of 10 cm/s are presented in Table 1.

### 3.2. ECST Criteria

**Figure 6:**
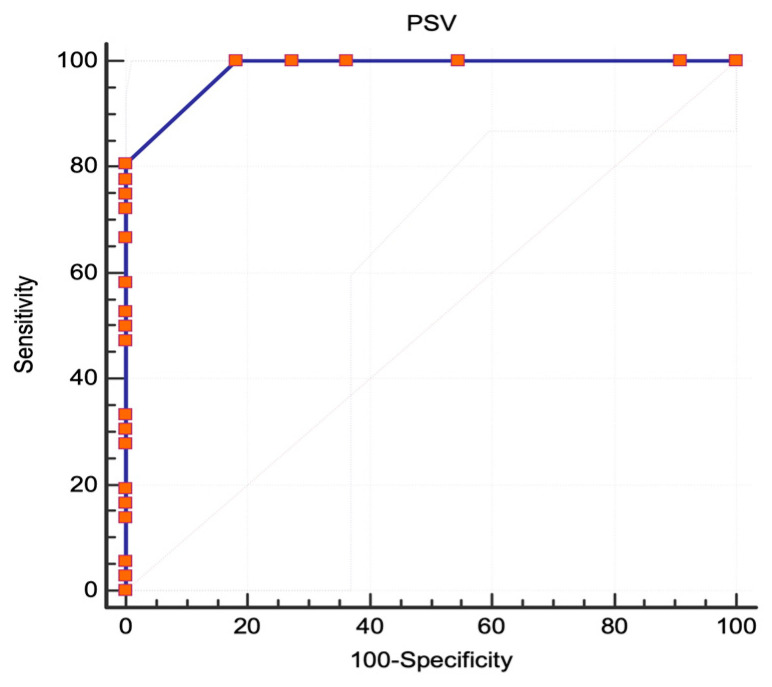
ECST criteria, ROC analysis. The area under the curve for stenoses ≥80%; Abbreviations: ECST, European Carotid Surgery Trial [7]; ROC, receiver operating characteristic; PSV, peak systolic velocity.

In this study, the ECST angiographic protocol was used to examine 47 lesions. Out of these, 36 lesions (76.5%) were found to be severe (≥80%). The AUC was 0.982 (95% CI 0.893 to 1, standard error 0.0133, *p* < 0.0001), showing an excellent correlation between PSV and severe ICA stenoses (Figure 6). The best PSV value to determine severe stenosis was found to be 180 cm/s. This criterion had a sensitivity of 100% (95% CI 90.3–100) and a specificity of 81.82% (95% CI 48.2–97.7). The diagnostic test was found to perform well with a Youden index of 0.818. Another threshold value of 200 cm/s was also found to have a similar statistical power with a sensitivity of 77.78% (95% CI 60.8–89.9) and a specificity of 100% (95% CI 71.5–100). The sensitivity and specificity of various PSV thresholds are shown in Table 1.

## 4. Discussion

The Society of Radiologists in Ultrasound [12] has developed recommendations for diagnosing and stratifying carotid stenoses. A stenosis of more than 50% in the ICA is identified if the PSV is ≥125 cm/s and sonographically visible plaque is present. A stenosis of ≥70% in ICA is diagnosed when PSV is greater than 230 cm/s, with visible luminal narrowing. However, the guideline does not provide specific PSV thresholds for ≥60% ICA stenosis, which may be helpful in certain clinical scenarios.

CEA has a significant benefit in patients with symptomatic NASCET ICA stenoses ≥70% (except true total vessel occlusion) [6] and a small benefit in patients with symptomatic 50–69% NASCET ICA stenoses [21]. For asymptomatic patients, a small benefit was found for CEA in ≥60% NASCET ICA stenoses [3,8,16,22]. CAS was found to be non-inferior to CEA in high-risk patients with symptomatic NASCET ICA stenoses ≥50% [23]. In the ECST trial, CEA benefit was found in lesions ≥80% [7]. The primary difference between the two thresholds (NASCET 70% and ECST 80%) lies in the different methods used to measure the stenosis. Both trials considered the narrowest lumen of the stenosis. The NASCET trial compared it to the vertical segment of the ICA, and the ECST trial compared it to the estimated carotid bulb diameter. Therefore, an 80% stenosis measured using the ECST method is similar to a 60–65% stenosis measured using the NASCET method.

The present study indicates that DUS is an effective diagnostic tool in evaluating carotid disease, but it must be interpreted in the clinical context of the patient. For patients with severe NASCET stenoses (≥70%), PSV was found to have high sensitivity and specificity. This study suggests that a PSV threshold of 200 cm/s is the best criterion for identifying severe stenoses, which is lower than the 230 cm/s PSV cut-off recommended by guidelines [3,12,16]. The NASCET trial [6] had previously reported that a PSV of 250 cm/s was the cut-off for severe stenoses. However, the present study found that the validated PSV cut-offs were too high for our vascular center. Other papers with similar protocols have reported the same 200 cm/s PSV threshold for ≥70% ICA stenoses [24,25]. Another paper reported that a PSV of ≥200 cm/s was the most reliable predictor of ≥70% ICA stenoses [26].

In 2005, a systematic review and meta-analysis of the association between DUS parameters and the degree of ICA stenosis were published [27]. The meta-analysis showed that a PSV of ≥200 cm/s had a sensitivity of 90% and a specificity of 94% in identifying ≥70% NASCET ICA stenoses. The present study found nearly identical results regarding the PSV threshold of 200 cm/s with a sensitivity of 90.32% and a specificity of 93.75%. Additionally, this meta-analysis reported that a PSV cut-off of 250 cm/s had a sensitivity of 76% and a specificity of 93%. Revascularization based on DUS protocols with PSV sensitivity as low as 75% and specificity lower than 85% were associated with overall harm [28]. Consequently, DUS calibration to other imaging techniques should be mandatory and regularly performed [29].

For patients with ≥50% NASCET ICA stenoses, PSV had low sensitivity. Histological studies suggest that PSV underestimates moderate lesions and overestimates tight lesions [30]. It is worth noting that PSV has some limitations. These include the presence of tandem lesions, the discrepancy between visual assessment of plaque and ICA PSV, elevated CCA velocity, hyper-dynamic cardiac state, low cardiac output, or calcified plaques with acoustic shadowing [12]. To overcome PSV limitations, medical specialists should consider additional parameters such as the ICA/CCA PSV ratio and ICA EDV. Repeating measurements and averaging the results can also help in minimizing the impact of measurement variability. Additionally, incorporating other imaging modalities such as magnetic resonance angiography (MRA) or computed tomography angiography (CTA) can provide additional information for accurate diagnosis [12,31].

The ICA/CCA PSV ratio and ICA EDV are used to evaluate the severity of stenoses in vessels or arteries. When stenoses are non-significant, with less than 50% blockage, the ICA/CCA PSV ratio is lower than 2.0 and the ICA EDV is below 40 cm/s. In moderate stenoses (50–69%), the ICA/CCA PSV ratio is between 2.0 and 4.0, and the ICA EDV ranges from 40 to 100 cm/s. For severe stenoses (≥70%), the ICA/CCA PSV ratio is above 4.0, and the ICA EDV is more than 100 cm/s [12,31,32].

However, these parameters also have several limitations. The ICA/CCA PSV ratio assumes that the CCA maintains a consistent diameter along its length, which may not always be the case. Additionally, the ratio does not account for variations in flow characteristics and vessel compliance. Therefore, relying solely on the ICA/CCA PSV ratio may lead to inaccurate assessments of stenosis severity. The measurement of EDV assumes a constant diastolic flow pattern, which may not be accurate in the presence of tandem lesions or other hemodynamic abnormalities. Moreover, fluctuations in heart rate and blood pressure can affect the accuracy of EDV measurements [12,32].

Another possible explanation is that the diagnostic accuracy of PSV for ICA stenosis quantification decreases when there is a contralateral tight lesion or occlusion. This causes an increase in volume through the ICA, resulting in an increased PSV and subsequent stenosis overestimation [33]. However, other data report a non-significant PSV variability with contralateral severe disease [24]. Sixty percent of the patients included had bilateral ≥50% ICA stenoses, which is considerably more than in similar papers [24,25,26].

On the other hand, asymmetrical stenosis could be overestimated by DSA since angiographic measurements are made on the incidence with the tightest residual lumen [28,34]. In current guidelines [3,12,16], a PSV ≥ 125 cm/s has a sensitivity of 98% and a specificity of 88% in identifying ≥50% NASCET ICA stenoses. Other studies have reported a good correlation between PSV and ≥50% ICA stenoses [24]. Finally, our study suggests that PSV values between 125 and 200 cm/s should be double-checked by other imaging modalities before making a final decision.

CTA is a non-invasive imaging technique that uses X-rays and computer algorithms to create detailed images of blood vessels. It provides high-resolution images of the carotid arteries, allowing for the assessment of stenosis severity, plaque morphology, and the presence of any associated complications. CTA has proven to be highly accurate in detecting carotid artery disease, with a sensitivity and specificity exceeding 90% [35]. MRA is another imaging modality that provides detailed images of the carotid arteries without using X-rays. It uses a powerful magnetic field and radio waves to generate high-resolution images. MRA offers several advantages over CTA, particularly in assessing plaque composition and identifying vulnerable plaques, which are at higher risk of causing strokes [36]. By incorporating these imaging modalities into clinical practice, healthcare professionals can enhance patient care and improve surgical outcomes in carotid artery disease.

In this study, a PSV of 180 cm/s was the best threshold for predicting ≥60% NASCET ICA stenoses. Other studies have reported that a PSV of 160 cm/s is the best-associated criterion [24]. However, there are limited data regarding these specific lesions since they were included in asymptomatic patient trials.

When the ECST angiographic protocol was used, the peak systolic velocity (PSV) was found to be significantly correlated with stenoses greater than 80%. It should be noted that a 60% NASCET stenosis is similar to an 80% ECST stenosis [37]. As a result, similar PSV values were found between NASCET 60% ICA stenoses and ECST 80% ICA stenoses. However, there are situations where the ECST angiographic protocol has significant advantages over the NASCET criteria. The NASCET method is not effective in measuring patients with extensive plaques within dilated carotid bulbs because the residual luminal diameter may be only slightly less than that of the distal ICA. In such cases, the NASCET measurement method will record non-significant stenosis, whereas the ECST method will measure this as a tight lesion [3]. This is another reason why it is suggested to use the 180 cm/s PSV cut-off for severe ECST lesions. A study reported a PSV threshold of 370 cm/s for severe ECST ICA stenoses [35]. However, the study correlated PSV to CTA findings, which cannot be directly compared to DSA.

In the past, when ECST [7] and NASCET [6] were randomizing patients, everyone underwent invasive angiography. However, invasive DSA is now less commonly used due to the potential risk of peri-procedural ischemic stroke. DSA is the most accurate tool when it comes to discordant non-invasive imaging results or when there is additional intracranial vascular disease [3,16]. Our analysis is a comparison of DUS properties with the ‘gold standard’ rather than with pathologic findings. We recognize that inter-observer variation can also be problematic for the ‘gold standard’ DSA test itself. Comparison with angiography is useful and justified because of the clinical situation of the selected patients. Moreover, influential trials documenting the efficacy of carotid revascularization (CEA or CAS) have either used invasive DSA or DUS calibrated to invasive angiography to define study participants [6,7].

Our study’s strength is the short time frame between the DUS examination and DSA acquisition, which adds to the accuracy of the data. We also took into account both NASCET and ECST angiographic classifications, which helped us better understand carotid artery disease pathophysiology. However, there are several limitations to our research. Firstly, being a single-center study, the results may be specific to our techniques and medical equipment. Secondly, the small number of carotid lesions, particularly for certain sub-groups, is likely to under-power the study. Thirdly, our cohort was selected based on the clinical need for DSA and is not representative of all patients with possible symptomatic carotid artery disease. Lastly, inter-observer variability in the DUS or DSA protocols was not assessed. Nonetheless, inter-observer errors tend to be systematic, and as long as the measurements and equipment are the same, the results in a given population are consistent and reproducible. Therefore, each medical center must establish local protocols and criteria for grading carotid stenoses, meaning a rigid PSV cut-off does not work for different vascular laboratories [38].

## 5. Conclusions

The PSV is an effective and straightforward marker for identifying severe stenoses, regardless of the angiographic protocol used. For ≥70% NASCET ICA stenoses, a PSV cut-off of 200 cm/s was found to be the best criterion, while a threshold of 180 cm/s was the best for ≥80% ECST ICA stenoses. Additionally, a PSV threshold of 180 cm/s was found to be associated with ≥60% NASCET ICA stenoses. However, PSV did not show a significant correlation with ≥50% NASCET ICA stenoses. In such cases, complementary imaging techniques like CTA, MRA, or DSA should be used in conjunction with DUS.

## Figures and Tables

**Table 1:** Sensitivity and specificity for different PSV values for detecting NASCET and ECST stenoses. * marks the most effective threshold, according to Youden’s index. Abbreviations: NASCET, North American Symptomatic Carotid Endarterectomy Trial [6]; ECST, European Carotid Surgery Trial [7]; ICA, internal carotid artery; PSV, peak systolic velocity.

PSV (cm/s)	Sensitivity	95% CI	Specificity	95% CI
NASCET protocol, ICA stenosis ≥ 60%				
170	100.00	90.3–100.0	63.64	30.8–89.1
180 *	100.00	90.3–100.0	72.73	39.0–94.0
190	100.00	90.3–100.0	81.82	48.2–97.7
200	80.56	64.0–91.8	100.00	71.5–100.0
210	77.78	60.8–89.9	100.00	71.5–100.0
220	75.00	57.8–87.9	100.00	71.5–100.0
NASCET protocol, ICA stenosis ≥ 70%				
180	100.00	88.8–100.0	50.00	24.7–75.3
190	100.00	88.8–100.0	56.25	29.9–80.2
200 *	90.32	74.2–98.0	93.75	69.8–99.8
210	87.10	70.2–96.4	93.75	69.8–99.8
220	83.87	66.3–94.5	93.75	69.8–99.8
230	83.87	66.3–94.5	100.00	79.4–100.0
ECST protocol, ICA stenosis ≥ 80%				
160	100.00	90.3–100.0	63.64	30.8–89.1
170	100.00	90.3–100.0	72.73	39.0–94.0
180 *	100.00	90.3–100.0	81.82	48.2–97.7
190	80.56	64.0–91.8	100.00	71.5–100.0
200	77.78	60.8–89.9	100.00	71.5–100.0
210	75.00	57.8–87.9	100.00	71.5–100.0

## Data Availability

Data are contained within the article.
